# Immunomodulatory Activity and Partial Characterisation of Polysaccharides from *Momordica charantia*

**DOI:** 10.3390/molecules190913432

**Published:** 2014-08-29

**Authors:** Yuan-Yuan Deng, Yang Yi, Li-Fang Zhang, Rui-Fen Zhang, Yan Zhang, Zhen-Cheng Wei, Xiao-Jun Tang, Ming-Wei Zhang

**Affiliations:** 1 State Key Laboratory of Phytochemistry and Plant Resources in West China, Kunming Institute of Botany, Chinese Academy of Science, Kunming 650201, China; E-Mail: yuanyuan_deng@yeah.net; 2 Sericulture and Agri-Food Research Institute, Guangdong Academy of Agricultural Sciences, Guangzhou 510610, China; E-Mails: yiyang1986@msn.com (Y.Y.); zlf6662@126.com (L.-F.Z.); ruifenzhang@163.com (R.-F.Z.); zhang__yan_@126.com (Y.Z.); zhencheng_wei@163.com (Z.-C.W.); xjtang66@163.com (X.-J.T.); 3 University of Chinese Academy of Science, Beijing 100049, China

**Keywords:** *Momordica charantia*, polysaccharide, immunomodulatory activity, structure

## Abstract

*Momordica charantia* Linn. is used as an edible and medicinal vegetable in sub-tropical areas. Until now, studies on its composition and related activities have been confined to compounds of low molecular mass, and no data have been reported concerning the plant’s polysaccharides. In this work, a crude polysaccharide of *M. charantia* (MCP) fruit was isolated by hot water extraction and then purified using DEAE-52 cellulose anion-exchange chromatography to produce two main fractions MCP1 and MCP2. The immunomodulatory effects and physicochemical characteristics of these fractions were investigated *in vitro* and *in vivo*. The results showed that intragastric administration of 150 or 300 mg·kg^−^·d^−1^ of MCP significantly increased the carbolic particle clearance index, serum haemolysin production, spleen index, thymus index and NK cell cytotoxicity to normal control levels in cyclophosphamide (Cy)-induced immunosuppressed mice. Both MCP1 and MCP2 effectively stimulated normal and concanavalin A-induced splenic lymphocyte proliferation *in vitro* at various doses. The average molecular weights of MCP1 and MCP2, which were measured using high-performance gel permeation chromatography, were 8.55 × 10^4^ Da and 4.41 × 10^5^ Da, respectively. Both fractions exhibited characteristic polysaccharide bands in their Fourier transform infrared spectrum. MCP1 is mainly composed of glucose and galactose, and MCP2 is mainly composed of glucose, mannose and galactose. The results indicate that MCP and its fractions have good potential as immunotherapeutic adjuvants.

## 1. Introduction

*Momordica charantia* Linn. is a typical sub-tropical vegetable belonging to the *Cucurbitaceae* family and is widely used as a traditional remedy for many diseases in Asia, Africa and South America. Many studies have confirmed that the fruit of *M.* *charantia* and its extracts possess antidiabetic/hypoglycaemic [1], hypolipidaemic [2], antiobesity [3], anti-inflammatory [4], antioxidant [5], antiviral [6,7], and antitumour [8,9] activities *in vivo* and *in vitro* and have no-to-low side effects in animals and in humans [10]. The activities have mainly been attributed to proteins/peptides [1,7,9], charantins [1], alkaloids [1] triterpenoids [3], phenolic compounds [5], and polysaccharides [11].

Immunity is related to the development and advance of many diseases. For instance, immunity is linked with obesity and insulin resistance. Obesity leads to adipocyte hypertrophy, which subsequently reduces the anti-inflammatory immune cells. As a result, the infiltration of proinflammatory cytokines eventually leads to the development of insulin resistance [12]. Further, accumulating evidence indicates that the innate and adaptive immune systems make crucial contributions to antitumour effects. Some antitumour agents can promote host immune system activation, resulting in enhanced antitumour responses [13]. *M. charantia* exhibits several of the aforementioned functional properties; however, immunomodulatory activity has not yet been observed. We hypothesised that many of the effects of this fruit might result from immunomodulation; hence, the study of immunomodulation by *M. charantia* is essential.

Polysaccharides isolated from botanical sources, such as *Angelica sinensis* [14], *Ganoderma lucidum* [15] and *Thamnolia vermicularis* var. *subuliformis* [16], exhibit excellent immunomodulating activities. As biological response modifiers, immunomodulatory polysaccharides do not cause harm and additional stress on the body, but rather help the body to adapt to environmental and biological stresses [17]. The activity of polysaccharides depends on their monosaccharide composition, molecular weight, and chain conformation [18]. It has been reported that highly branched water-soluble heteropolysaccharides obtained from *Rhizoma panacis Japonici* act as immunopotentiators to inhibit S-180 tumour cell growth in BALB/c mice. Such bioactivity might be due to unique structural features, including an α-(1→4)-d-glucan main chain and water-soluble galactose and mannose side chains [19]. Ke also reported that the stimulating activity of polysaccharides obtained from *Streptococcus equi* subsp*. Zooepidemicus* on splenocyte proliferation and acid phosphatase activity in peritoneal macrophages were related to the amount of chain branching as well as the amounts of arabinose, mannose and galactose present [20]. However, Mizuno indicated that the antitumour activity of polysaccharides obtained from mushrooms was strongly dependent on compounds with a high molecular weight, ranging from 500 kDa to 2000 kDa, rather than on compounds containing glycosidic bonds, such as (1→3)-β-glucans [21]. Therefore, evaluation of the immunoregulatory activity and structure of the polysaccharides present in *M.* *charantia* is important in the elucidation of their function and in their utilisation. In this paper, we provide a detailed report of the *in vitro* and *in vivo* immunostimulatory activities of the crude polysaccharide (MCP) and purified fractions (MCP1 and MCP2) of *M.* *charantia*. Furthermore, we also present the structural characterisation of MCP1 and MCP2 by gas chromatography, mass spectrometry (GC-MS), high-performance gel permeation chromatography (HPGPC) and Fourier transform-infrared (FTIR) spectroscopy.

## 2. Results and Discussion

### 2.1. Immunomodulatory Activity of MCP in Vivo

#### 2.1.1. Effects of MCP on Spleen and Thymus Indexes

**Figure 1:**
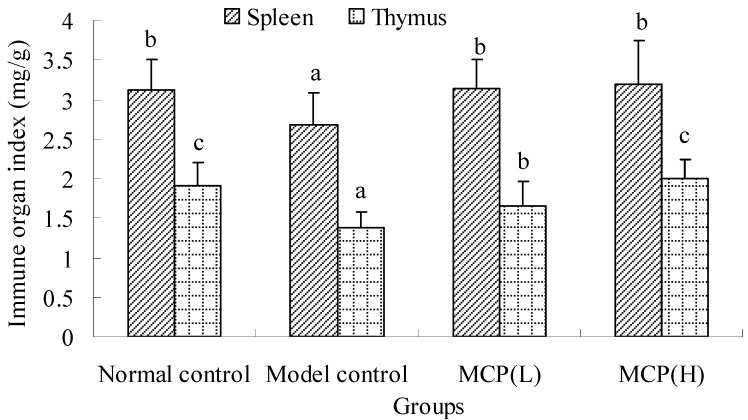
Effects of MCP on spleen and thymus indexes in immunosuppressed mice. The spleen/thymus index was measured as the ratio of the spleen/thymus weight (mg) to bodyweight (g). Values are presented as means ± SD (n = 12). The statistical significance of differences among the groups was evaluated using ANOVA, followed by the S-N-K test. Different letters for same index among the groups represent significant differences at *p* < 0.05.

The immunomodulatory activity of MCP was evaluated in immunosuppressed mice. As seen in Figure 1, the spleen and thymus indexes in the cyclophosphamide (Cy)-treated model control group were significantly lower than those in the normal control group (*p* < 0.05). These results suggested that Cy caused spleen and thymic atrophy. The differences between the spleen indexes of the normal control and MCP groups were not significant (*p* > 0.05). The thymus indexes of the animals treated with MCP were significantly higher than those of the model controls. The thymus index of the MCP(H) group was higher than that of the MCP(L) group and showed no obvious differences from the normal control group (*p* > 0.05). These results indicated that MCP could maintain the normal morphology of immune organs.

#### 2.1.2. Effect of MCP on Macrophage Phagocytosis

The *in vivo* clearance rate of carbon particles from the mice increased exponentially with the blood concentration of carbon. Macrophage phagocytosis is expressed as the carbolic particle clearance index (α) (Table 1), which reflects the function of macrophages. The α value of the model control was significantly lower than that of the normal control (*p* < 0.05). After administration with MCP, the phagocytic function of the Cy-immunosuppressed groups was statistically stronger that of the model control (*p* < 0.05) and was not significantly different from that of the normal control (*p* > 0.05). Moreover, the α values did not significantly differ between the MCP groups (*p* > 0.05). These results suggested that MCP could increase macrophage phagocytosis to a normal level in immunosuppressed mice.

**Table 1:** Effects of MCP on carbolic particle clearance (α), serum haemolysin production (HC_50_), spleen lymphocyte proliferation and NK cell activity in immunosuppressed mice. The data are presented as the means ± SD (n = 12). The statistical significance of differences among the groups was evaluated using ANOVA, followed by the S-N-K test. Different letters for the same index indicate significant differences at *p* < 0.05.

Group	Dose (mg·kg^−1^·d^−1^)	α	HC_50_	PI	NK Activity (%)
**Normal control**	—	5.53 ± 0.95 ^b^	144.33 ± 11.07 ^b^	1.001 ± 0.107 ^b^	43.757 ± 5.160 ^b^
**Model control**	—	4.22 ± 0.64 ^a^	123.97 ± 9.53 ^a^	0.659 ± 0.127 ^a^	35.382 ± 9.152 ^a^
**MCP(L)**	150	4.97 ± 0.67 ^b^	136.04 ± 9.02 ^b^	1.087 ± 0.274 ^b^	43.478 ± 8.325 ^b^
**MCP(H)**	300	5.04 ± 0.42 ^b^	158.98 ± 8.93 ^b^	1.396 ± 0.204 ^c^	46.397 ± 7.937 ^b^

#### 2.1.3. Effect of MCP on Haemolysin Production

Chicken red blood cells (CRBC) are phagocytosed by peritoneal macrophages *in vivo*, and the index reflects murine humoural immune function. The response of the serum haemolysin content to CRBC, which is expressed by the HC_50_ values shown in Table 1, was used to evaluate the effect of MCP on the complement system of immunosuppressed mice. The HC_50_ values indicate that the production of serum haemolysin in mice was obviously decreased as a result of the immunosuppression treatment (*p* < 0.05) and that MCP significantly enhanced the production of serum haemolysin in model mice to a level that is statistically comparable to the normal level. These results suggest that MCP could obviously increase the humoural immunity of immunosuppressed mice.

#### 2.1.4. Effect of MCP on Splenic Lymphocyte Proliferation

Splenic lymphocytes of immunosuppressed mice were isolated and cultured. The proliferation of splenic lymphocytes induced by ConA was used to confirm the effect of MCP on the cellular immune response. The PI values shown in Table 1 indicate that the splenic lymphocyte proliferation in the model control was significantly lower than that in the normal control and MCP groups (*p* < 0.05). The PI value of the MCP(L) group is statistically comparable to that of the normal control group (*p* > 0.05), and both of these values are lower than that of the MCP(H) group (*p* < 0.05). These results suggested that MCP could increase the cellular immunity of immunosuppressed mice.

#### 2.1.5. Effect of MCP on NK Cell Cytotoxicity

Splenic lymphocytes of immunosuppressed mice were isolated to evaluate the effect of MCP on NK cell cytotoxicity. Tumour cell elimination is known to be partly mediated by the NK cell cytotoxicity, which can be evaluated based on splenocyte cytotoxicity against NK-sensitive tumour cells (YAC-1), as shown in Table 1. NK cell cytotoxicity was significantly suppressed in the model control compared with the normal control (*p* < 0.05). The values of NK cell cytotoxicity in both MCP groups reached the level seen for the normal control group (*p* > 0.05). These results suggest that MCP could improve NK cell vitality in immunosuppressed mice.

Cy is a widely used cancer chemotherapy agent; however, it causes toxicity in several organs including the spleen, thymus, and liver. Immunosuppressant activity represents one of the most difficult clinical challenges. It has been reported that Cy can suppress humoural and cellular immune function through lymphocyte depletion and macrophage deficiency [22]. In the present work, an immunological evaluation based on the immune organ index, macrophage phagocytosis, serum haemolysin production, splenic lymphocyte proliferation and NK cell cytotoxicity indicated that immunosuppression induced by Cy was effective. After administration of 150 or 300 mg·kg^−1^·d^−1^ of MCP for 30 days, all immunological parameters were restored to the normal level and were comparable to those in immunosuppressed mice treated with longan polysaccharide in the dose range of 50 to 200 mg·kg^−1^·d^−1^ [23]; thus, MCP has potential application as a natural immunomodulating agent.

Phagocytes (neutrophils, monocytes and macrophages) are key participants in the innate immune response; in this response, macrophages and neutrophils represent the first line of host defence after the epithelial barrier. Phagocytic function represents the non-specific immune status of animals. The αvalues obtained in the test support the notion that MCP might initiate the mononuclear phagocytic system (MPS) function of the immune reaction against foreign materials [24]. MCP significantly enhanced the non-specific immune function in Cy-treated mice and promoted the development of the spleen and thymus in immunosuppressed mice to the normal level. Treatment with MCP *in vivo* promoted the proliferation of spleen lymphocytes to ConA, indicating that MCP affected the recovery and enhancement of T cell activity [15]. MCP also significantly increased the cytolytic response against CRBC, and this might partly have occurred *via* the stimulation of antibody-secreting B-cells in the spleen [23,25]. It has been suggested that MCP exerts a dual synergistic stimulation of B and T lymphocytes [24]. NK cells play an important role in immunoregulation and tumour surveillance as anti-tumour effector cells. MCP accelerated the recovery of splenic NK cell numbers in Cy-treated mice, thus strengthening the cytotoxicity to Yac-1 tumour cells. As presented above, polysaccharides from *M. charantia* can promote the compensation of immune cell deficiency and enhance immune cell function to improve Cy-induced immunosuppression.

### 2.2. Effects of MCP1 and MCP2 on Splenic Lymphocyte Proliferation in Vitro

The immunomodulatory activity of MCP1 and MCP2 was evaluated in primary cultured splenic lymphocytes obtained from normal mice. As seen in Table 2, MCP1 and MCP2 can stimulate splenic lymphocyte proliferation in a dose-dependent manner. MCP1 obviously promoted the proliferation of splenic lymphocytes at various doses except 60 µg/mL, and the highest proliferation index was found at 180 µg/mL, at 3.82 times the value of the normal group. However, the value was not significantly different from that obtained at 100 µg/mL (*p* > 0.05). The proliferation-promoting activities of MCP2 on splenic lymphocytes were effectively promoted in the dose range of 20 to 100 µg/mL (*p* < 0.05), and the effects of these doses did not significantly differ between groups (*p* > 0.05). Moreover, 100 µg/mL of MCP1 stimulated the proliferation of splenic lymphocytes significantly more than the same dose of MCP2 (*p* < 0.05).

**Table 2:** Effects of MCP1 and MCP2 on the proliferation of splenic lymphocytes *in vitro*. Proliferation was assessed using the MTT assay, and the results are expressed as means ± SD(n = 6). Different letters (a, b, c, d, e) indicate a significant difference between concentrations of individual polysaccharides by ANOVA followed by the S-N-K test (*p* < 0.05). The statistical significance of differences between polysaccharides at the same concentration was evaluated using the *T*-test. * indicates a significant difference (*p* < 0.05) between MCP2 and MCP1 at the same concentration. ^∆^ indicates a significant difference (*p* < 0.05) between MCP1+ConA and MCP1 at the same concentration. ^#^ indicates a significant difference (*p* < 0.05) between MCP2+ConA and MCP2 at the same concentration.

Dose (µg/mL)	MCP1	MCP2	MCP1+ConA	MCP2+ConA
**0**	1.00 ± 0.16 ^a^	1.00 ± 0.16 ^a^	1.67 ± 0.34 ^a∆^	1.67 ± 0.34 ^a^^#^
**20**	2.00 ± 0.69 ^bc^	2.18 ± 0.47 ^b^	3.29 ± 0.38 ^b∆^	2.86 ± 0.36 ^b^
**60**	1.73 ± 0.31 ^ab^	2.18 ± 0.47 ^b^	3.36 ± 0.39 ^bc∆^	2.32 ± 0.55 ^b^
**100**	3.18 ± 0.42 ^de^	2.11 ± 0.34 ^b^*	4.08 ± 0.38 ^c∆^	2.32 ± 0.27 ^b^
**140**	2.82 ± 0.42 ^cd^	2.27 ± 0.63 ^b^	2.82 ± 0.08 ^b^	2.77 ± 0.31 ^b^
**180**	3.82 ± 0.72 ^e^	2.82 ± 0.57 ^b^	2.77 ± 0.57 ^b^	3.64 ± 0.28 ^c^

The induction of spleen lymphocyte proliferation by ConA *in vitro* was used to evaluate T lymphocyte activity [26], and proliferation was significantly induced by ConA compared with the normal control group (*p* < 0.05). Treatment with MCP1 significantly stimulated ConA-induced proliferation at all doses (*p* < 0.05), and treatment with the dose of 100 µg/mL resulted in the highest proliferation index, presenting obvious differences from other doses, with the exception of 60 µg/mL (*p* < 0.05). Likewise, the significant proliferation-promoting effects of MCP2 in the dose range of 20 to 140 µg/mL did not significantly differ between groups, but the proliferation obtained using this dose range was lower than that observed when using a dose of 180 µg/mL (*p* < 0.05). Furthermore, 20, 60 and 100 µg/mL of MCP1 also significantly stimulated ConA-induced proliferation compared with the same dose of MCP1 alone (*p* < 0.05), but MCP2 did not significantly improve the stimulation when combined with ConA at any dose.

We evaluated the immunomodulatory effects of MCP1 and MCP2 on murine splenocytes *in vitro*. ConA activated T lymphocytes by binding to the specific receptor on T lymphocyte cell membranes [27]. MCP1 and MCP2 effectively promoted normal and ConA-induced splenic lymphocyte proliferation at a certain dose in the range of 20 to 180 µg/mL; thus, the mechanism involved might be related to the activation of T cells via the binding of the polysaccharides to receptors specifically expressed on T cells [28]. In addition, the promotion of ConA-induced splenic lymphocyte proliferation by MCP1 was comparable to that by *Pleurotus ostreatus* polysaccharide POP [29], and both of these compounds resulted in the highest proliferation index at 100 µg/mL. However, MCP2 and polysaccharides from *Armillaria mellea* [30] and *Codonopsis pilosula* [31], which did not obviously differ from each other with respect to proliferation-stimulating activity, resulted in the highest index at the higher dose.

### 2.3. Isolation and Purification of MCP

**Figure 2:**
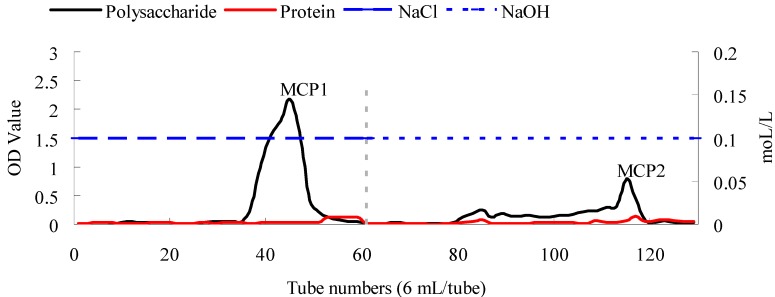
DEAE-52 cellulose anion-exchange chromatogram of the elution of crude polysaccharides from *M. charantia* polysaccharides using 0.1 mol/L NaCl and 0.1 mol/L NaOH. Polysaccharides and protein were detected using the phenol-sulphuric acid method (measured at 490 nm) and UV measurement (at 280 nm), respectively.

The two main fractions eluted from an anion-exchange column using 0.1 mol/L NaCl and 0.1 mol/L NaOH were named MCP1 and MCP2 (Figure 2), respectively, and their yields accounted for 48.74% and 15.16% of the crude polysaccharide MCP. Based on the elution conditions, it was suggested that these fractions comprised acid polysaccharides [32]. The polysaccharide contents of MCP, MCP1 and MCP2 were 62.76%, 84.03% and 72.57%, respectively, and the protein contents of MCP, MCP1 and MCP2 were 4.62%, 2.08% and 6.87%, respectively.

### 2.4. Characterisation of MCP1 and MCP2

The monosaccharide compositions of MCP1 and MCP2 exhibited obvious differences, as seen in Table 3. MCP1 mainly contained glucose and galactose, and MCP2 contained glucose, mannose and galactose. The average molecular weights (*M*_w_) of the fractions were 85.5 kDa and 441 kDa (retention times: 12.32 and 18.69 min), respectively. The polydispersion indexes were individually determined to be 4.275 and 3.267, indicating the inhomogeneity of each fraction.

The FTIR spectra of MCP1 and MCP2, including the absorption, functional group and structural characteristics, are summarised in Table 4. Both MCP1 and MCP2 exhibited features that are characteristic of polysaccharides, including hydroxyl group bands, alkyl group bands and carboxyl group bands [33,34,35]. Absorption peaks at 1240.7 cm^−1^ were assigned to the stretching vibrations of the S=O bond, providing evidence of the existence of a sulphate ester, indicating that MCP1 is a sulphated polysaccharide [36]. The bands at 1145.4 and 1099.6 cm^−1^ were assigned to the valent vibrations of the C–O–C bond and the glycosidic bridge of MCP1 [37]. Moreover, characteristic absorption bands at approximately 955, 894 and 835 cm^−1^ indicated the existence of a pyranoid ring, β-d-glucopyranose and α-d-glucopyranose, respectively, in MCP1 and MCP2 [33,35,38]. These findings indicate that both MCP1 and MCP2 mainly comprised d-glucosyl residues in the pyranose form, although α- and β- types were simultaneously present. These data suggest that both MCP1 and MCP2 are heteropolysaccharides based on a glucan backbone.

**Table 3:** Monosaccharide compositions of MCP1 and MCP2. The monosaccharide compositions of MCP1 and MCP2 were determined using gas chromatography–mass spectrometry.

Composition	MCP1		MCP2	
	Retention Time (min)	Molar Ratio	Retention Time (min)	Molar Ratio
**Ribose**	5.97	1.00	5.95	1.86
**Rhamnose**	6.05	6.33	6.03	1.00
**Arabinose**	6.11	9.07	6.14	8.92
**Xylose**	6.25	3.78	6.23	9.62
**Mannose**	8.05	4.71	8.05	34.18
**Glucose**	8.12	27.28	8.12	44.20
**Galactose**	8.55	19.58	8.33	23.61

**Table 4:** FTIR spectrum analysis of the functional groups of MCP1 and MCP2. The FTIR spectra of MCP1 and MCP2 were determined using a FTIR spectrophotometer operating in the frequency range of 4000–400 cm^−1^.

Absorption (cm^−1^)		Functional Group	Structural Characteristic
MCP1	MCP2		
3431.7	3431.2	Hydroxyl group (–OH)	O–H stretching vibration
2931.9	2925.8	Alkyl group (–CH_2_-)	C–H stretching vibration
1618	1618.5	Carboxyl group (–C=O or –CHO)	C=O stretching vibration
1419.8	1412.7	Carboxyl group (–COOH)	C–O stretching vibration
1331.5		Carboxyl group (–COOH)	C=O symmetrical stretching vibration
1240.7		Sulphate group (–O–SO_3_–)	S=O stretching vibration of
1145.4, 1099.6		Ether (–C–O–C–)	C–O–C covalent vibration
1018.8	1025.8	Hydroxyl group (–OH)	O–H bending vibration
954.9	955.5	Pyranose ring	Rolling vibration at the end of the methine
894.2	894.7	β-d-Glucopyranose	
835.2	835.3	α-d-Glucopyranose	

Liu and co-workers reported the purification of an *M. charantia* polysaccharide fraction using DEAE-52 cellulose anion-exchange chromatography (eluted using 0.1 mol/L NaCl) and Superdex G-100; the product was a homogeneous polysaccharide with an average molecular weight of 93.58 kDa that contained pyranosides and a β-glycosidic bond [39]. The reported *M. charantia* polysaccharide fraction was purified under the same separation conditions as those used here for MCP1; thus, their product and MCP1 are of similar average molecular weight and have similar glucosidic bonds. Orlovskaya reported that the main monosaccharide component of the water-soluble polysaccharide isolated from *M. charantia* seeds was glucose and that the main monosaccharide components of a water-soluble polysaccharide isolated from pericarp were xylose and arabinose [40]. We found that MCP1 and MCP2, which were isolated from *M. charantia* fruits, mainly comprised glucose, galactose and mannose. Studies have shown that polysaccharides obtained from different *M. charantia* tissues have different monosaccharide compositions.

It is well known that several pharmacological properties of polysaccharides are correlated with their chemical composition and configuration. Although it is difficult to clarify the relationship between the structure and activity of complex polysaccharides, some possible correlations can be inferred. The α (1 → 4) glucan from *Tinospora cordifolia* has been reported to possess potent immunostimulatory effects, and the immunostimulating signalling mechanism of the (1,4)-α-d-glucan occurred through the activation of macrophages via TLR6 signalling, NF-κB translocation and cytokine production [41]. β-(1,3)-d-glucan is the active ingredient responsible for the stimulating effects of polysaccharopeptides on various immune subsets [42]. In this work, MCP1 and MCP2 were characterised as heteropolysaccharides constructed on a glucan backbone. These structural features may be partly responsible for the strong immunomodulatory effects of MCP1 and MCP2.

Furthermore, sulphate content and molecular weight are also regarded as important factors for the immunoregulation activities of polysaccharides. Polysaccharides containing sulphate present strong immunostimulatory activity that is greatly diminished when the polysaccharides are desulphated [43,44]. Zhang found that polysaccharides of low molecular weight exhibited stronger antitumour activity and that the Mw of polysaccharides might play a more important role than sulphate content [45]. Our results are consistent with the above reports. MCP1 stimulated the proliferation activity of spleen lymphocytes more than MCP2 *in* *vitro*, and this result might be due to the presence of sulphate in MCP1. Moreover, the molecular weights of MCP1 and MCP2 were approximately 85.5 kDa and 441 kDa, respectively. Low molecular weight might increase the opportunity of samples to recognise and bind to immune complexes, which would explain why MCP1 exhibits stronger immunomodulatory activity than MCP2. Taken together, these findings show that the sulphation and lower molecular weight of MCP1 might be responsible for its higher immunomodulatory activity.

## 3. Experimental Section

### 3.1. Materials and Chemicals

Fresh *M. charantia* (cv. Bilv No. 2) fruits, harvested in July 2012, provided by the Vegetable Research Institute of the Guangdong Academy of Agriculture Sciences (Guangzhou, China). Fresh fruits were split, and the seeds were manually removed. The fleshy parts of the fruits were then cut into small pieces and dried at 60 °C for 8 h (moisture content < 13%). The dry pieces were then ground in a mill (A11 basic, ZKA-Werke, Staufen, Germany), and the resulting powder was packaged and stored at −20 °C. DEAE-52 cellulose, glucose, galactose, arabinose, rhamnose, mannose, concanavalin A (ConA) and 3-(4,5-dimethylthiazol-2-yl)-2,5-diphenyltetrazolium bromide (MTT) were purchased from Sigma (St. Louis, MO, USA). Cyclophosphamide (Cy) was purchased from Heng Rui Medicine Co., Ltd. (Jiangsu, China). RPMI-1640 medium and new bovine calf serum were purchased from Gibco Life Technologies (Grand Island, NY, USA). Other chemicals used were analytical grade.

### 3.2. Preparation of M. charantia Polysaccharides

Dried *M. charantia* powder (50 g) was soaked in 80% ethanol (100 mL) to remove pigments and small lipophilic substances. The residue was then extracted with distilled water (1 L) at 95 °C for 4 h. The water-soluble extract was then filtered through Whatman No.1 paper, concentrated at 60 °C using a vacuum rotary evaporator (N-1100D-W/WD, Eyela, Tokyo, Japan), and deproteinated using the Sevag method [46]. Then, three volumes of dehydrated ethanol were added, and the solution was left overnight at 4 °C to precipitate the polysaccharides. The precipitate was centrifuged at 4500 rpm for 10 min and lyophilised, yielding a crude polysaccharide fraction (MCP). The extraction yield was 8.9%.

The MCP was fractionated according to our previous method with slight modifications [35]. MCP (200 mg) was redissolved in distilled water (40 mL) and centrifuged at 4500 rpm for 15 min. The supernatant was injected into an anion-exchange DEAE-52-cellulose column (500 mm × 26 mm), and minor polysaccharides were removed by elution with 0.05 mol/L NaCl. The column was then eluted with 0.1 mol/L NaCl and 0.1 mol/L NaOH for 8 h each time. Eluate (6 mL/tube) was collected at a flow rate of 0.4 mL/min. Polysaccharide concentration was determined using the phenol-sulphuric acid method [47]. Protein concentration was measured using Bradford’s method [48]. The tubes containing the same fraction were combined and concentrated at 55 °C using a vacuum rotary evaporator. The concentrated solution was lyophilised to recover the purified polysaccharides, which were then packaged and stored in a desiccator at room temperature.

### 3.3. Immunomodulatory Activity Analysis

#### 3.3.1. Animals and Cells

Specific pathogen-free Kunming mice (male, 20.0 ± 2.0 g, certificate number: SCXK-Yue 2011-0029) and the YAC-1 lymphoma cell line were obtained from the Experimental Animal Centre of Sun Yat-sen University (Guangzhou, China). The mice were housed under normal laboratory conditions (12 h light/12 h dark cycle, 60% humidity and 25 ± 1 °C) with free access to standard rodent chow and water. The animals were acclimatised to the laboratory for one week. Three batches of mice were bred for *in vivo* immunomodulatory evaluation, each of which contained four groups (twelve mice per group). These groups were treated as shown in Table 5. To induce immunosuppression, animals were injected intraperitoneally with cyclophosphamide at a dose of 80 mg·kg^−1^·d^−1^ for 3 days. The normal control group received vehicle only. MCP was dissolved in distilled water, and mice were intragastrically administered MCP at 150 or 300 mg·kg^−1^ bodyweight each day for 30 days. The control and model groups were administered distilled water. The *in vivo* immunomodulatory activities of the polysaccharides were tested at 33 days. In addition, 10-week-old mice were euthanised to collect cells for *in vitro* immunomodulatory evaluation. The experiments were approved by the Animal Care and Use Committee of Guangdong Province (Guangzhou, China) and performed according to the Laboratory Animal Management Regulations of Guangdong Province.

**Table 5:** Groups of mice undergoing different *in vivo* treatments.

Groups	Normal Saline (mL·kg^−^·d^−1^)	Cyclophosphamide (mg·kg^−1^·d^−1^)	Normal Saline (mL·kg^−1^·d^−1^)	MCP (mg·kg^−1^·d^−1^)
	Intraperitoneal Injection (Days 1–3)		Peroral Administration (Days 4–33)	
**Normal control**	5	–	5	–
**Model control**	–	80	5	–
**MCP(L)**	–	80	–	150
**MCP(H)**	–	80	–	300

#### 3.3.2. Macrophage Phagocytosis Assay

The phagocytosis of macrophages in liver and spleen was assessed based on the scavenging efficiency of vena-caudalis-injected carbon particles in blood [23,49] and expressed as the index of carbolic particle clearance (α). α was calculated using the following formula: α = body weight/(liver weight + spleen weight) [(lgA_1_ − lgA_2_)/(t_2_ − t_1_)]^1/3^, where A_1_ represents the absorbance of the blood sample at time t_1_ and A_2_ represents the absorbance of the blood sample at time t_2_; t_1_ and t_2_ were 2 and 10 min, respectively.

#### 3.3.3. Measurement of Serum Haemolysin

Mice were intraperitoneally immunised with CRBC at 27 days. The antibody production against CRBC was then measured as the 50% haemolytic complement (HC_50_) activity of serum using a spectrophotometric method [23,49] and was calculated as follows: HC_50_ = A_serum_/A_CRBC_ × n. A_serum_ represents the absorbance of the serum sample, A_CRBC_ represents that of the CRBC control, and n was 150, representing the dilution of the serum sample.

#### 3.3.4. Measurement of Spleen and Thymus Indexes

The spleen and thymus of mice were separated and weighed under aseptic condition at 33 days. The indices (mg·g^−1^) of spleen and thymus were calculated as the ratio of organ weight to body weight.

#### 3.3.5. Splenic Lymphocyte Proliferation Assay

The proliferation-promoting effects of MCP on splenic lymphocytes in immunosuppressed mice were performed on day 33. Splenic lymphocytes were isolated from immunosuppressed/normal mice and cultured. Next, these cells were measured according to our previously modified MTT method [23,35]. Stimulation of splenic lymphocytes by MCP1 and MCP2 in the dose range of 20–180 µg/mL *in vitro* was performed with another batch of 10-week-old mice. The *in vitro* and *in vivo* results were expressed as the proliferation index (PI), which was calculated using the following formula: PI = A_s_/A_c_ × 100%. A_s_ represents the absorbance of the stimulated/strengthened group, and A_c_ represents the absorbance of the control.

#### 3.3.6. Cytotoxicity Assay of NK Cells

NK-sensitive YAC-1 lymphoma cells were used as a target control, splenocytes were used as an effector control, and YAC-1 cells and splenocytes were mixed for use as the experimental group. The NK cell cytotoxicity against YAC-1 lymphoma cells was calculated as follows: NK activity = [A_T_ − (A_exp_ − A_E_)]/A_T_ × 100%, where A_T_, A_exp_ and A_E_ represent the absorbances of the experimental group, the effector control and the target control, respectively [23,50].

### 3.4. Characterisation of Polysaccharides

#### 3.4.1. Characterisation and Identification of Monosaccharides

The identification and quantification of the monosaccharide composition of the polysaccharides was performed using gas chromatograph-mass spectrometry according to our previous work [33]. Briefly, polysaccharide samples (40 mg) were hydrolysed using 2 mol/L sulphuric acid (10 mL) at 100 °C for 6 h. After neutralising the excess sulphuric acid with saturated barium hydrate, the hydrolysates and monosaccharide standards were derivatised with hydroxylamine hydrochloride. Then, all derivatives were analysed using GC–MS and a DB-1 column (15 m × 0.2 mm, 0.33 µm, J&W Scientific, Folsom CA, USA). The chromatography was performed using an Agilent 6890 GC coupled with a 5973 MS (Agilent Technologies, Santa Clara, California, USA) under the following conditions: initial temperature of column, 100 °C; the temperature was increased to 280 °C at a rate of 10 °C/min and maintained at 280 °C for 15 min; injection temperature, 280 °C. The sample was injected into the column using the split ratio of 10:1.

#### 3.4.2. Molecular Weight Measurement

The homogeneity and average M_W_ of the polysaccharides were determined using high-performance gel permeation chromatography [51]. The polysaccharide solution was applied to a Waters 600E HPLC (Millipore, Milford, MA, USA) instrument equipped with a TSK-GEL G3000 SWXL column (300 mm × 7.8 mm, TosoHass, Tokyo, Japan), eluted with 0.05 mol/L NaH_2_PO_4_-Na_2_HPO_4_ buffer (pH = 6.7, containing 0.05% NaNO_3_) at a flow rate of 0.5 mL/min and detected using a differential refractive index detector (Optilab rEX, Wyatt, Santa Barbara, CA, USA). Standards of known M_W_ (7.38 × 10^ 2^, 5.80 × 10^3^, 1.22 × 10^4^, 2.37 × 10^4^, 4.80 × 10^4^, 1.00 × 10^5^, 1.86 × 10^5^, 3.80 × 10^5^, and 8.35 × 10^5^ Da) were used as calibration standards to estimate the M_W_ of the polysaccharide sample.

#### 3.4.3. Fourier Transform Infrared Spectrum

Two milligrams of sample was ground with 200 mg of potassium bromide (spectroscopic grade) using a mortar and pestle. The FTIR spectrum in the frequency range 4000–400 cm^−1^ was obtained using a spectrophotometer (Nexus 5DXC FTIR, Thermo Nicolet, Waltham, MA, USA) [33].

### 3.5. Statistical Analysis

Data are expressed as the means ± standard deviations. The statistical significance of the differences between groups was evaluated using one-way ANOVA, followed by the Student-Newman-Keuls test, using SPSS 13.0 software. The statistical significance of differences between the two groups was evaluated using an independent-sample *T-*test. A *p*-value of 0.05 was used as the threshold for significance.

## 4. Conclusions

A polysaccharide from *M. charantia* (MCP) was isolated and purified using DEAE-52 cellulose anion-exchange chromatography. Two major components, MCP1 and MCP2, were obtained. The present study demonstrates for the first time that MCP can reverse immunosuppression in Cy-induced mice. Although the mechanism of MCP is unknown, based on the results, we conclude that MCP directly immunostimulates splenic lymphocytes and immunologically enhances the cellular and humoural immune functions of immunosuppressed mice effectively. In addition, MCP1 and MCP2 were found to effectively stimulate normal and ConA-induced splenic lymphocyte proliferation *in vitro*. The molecular weights of the two isolated fractions were 8.55 × 10^4^ and 4.41 × 10^5^ Da, respectively. MCP1 mainly comprised glucose and galactose, and MCP2 mainly comprised glucose, mannose and galactose. Both fractions were heteropolysaccharides based on a glucan backbone. This study suggests that MCP and its fractions can be used as efficacious immunostimulants to lessen chemotherapy-induced immunosuppression and in the production of functional foods and pharmaceuticals.
